# Associations Between Neighborhood Resources and Youths’ Response to Reward Omission in a Task Modeling Negatively Biased Environments

**DOI:** 10.1016/j.jaac.2024.05.011

**Published:** 2024-05-17

**Authors:** Berron Brown, Lynn T. Nguyen, Isaac Morales, Elise M. Cardinale, Wan-Ling Tseng, Cameron C. McKay, Katharina Kircanski, Melissa A. Brotman, Daniel S. Pine, Ellen Leibenluft, Julia O. Linke

**Affiliations:** National Institute of Mental Health, National Institutes of Health, Bethesda, Maryland.; National Institute of Mental Health, National Institutes of Health, Bethesda, Maryland.; National Institute of Mental Health, National Institutes of Health, Bethesda, Maryland.; The Catholic University of America, Washington, DC.; Yale School of Medicine, New Haven, Connecticut.; National Institute of Mental Health, National Institutes of Health, Bethesda, Maryland.; National Institute of Mental Health, National Institutes of Health, Bethesda, Maryland.; National Institute of Mental Health, National Institutes of Health, Bethesda, Maryland.; National Institute of Mental Health, National Institutes of Health, Bethesda, Maryland.; National Institute of Mental Health, National Institutes of Health, Bethesda, Maryland.; UTHealth, Houston, Texas, and the University of Freiburg, Germany.

**Keywords:** Child Opportunity Index (COI), reward omission, sadness, frustration, brain imaging

## Abstract

**Objective:**

Neighborhoods provide essential resources (eg, education, safe housing, green space) that influence neurodevelopment and mental health. However, we need a clearer understanding of the mechanisms mediating these relationships. Limited access to neighborhood resources may hinder youths from achieving their goals and, over time, shape their behavioral and neurobiological response to negatively biased environments blocking goals and rewards.

**Method:**

To test this hypothesis, 211 youths (aged ~ 13.0 years, 48% boys, 62% identifying as White, 75% with a psychiatric disorder diagnosis) performed a task during functional magnetic resonance imaging. Initially, rewards depended on performance (unbiased condition); but later, rewards were randomly withheld under the pretense that youths did not perform adequately (negatively biased condition), a manipulation that elicits frustration, sadness, and a broad response in neural networks. We investigated associations between the Childhood Opportunity Index (COI), which quantifies access to youth-relevant neighborhood features in 1 metric, and the multimodal response to the negatively biased condition, controlling for age, sex, medication, and psychopathology.

**Results:**

Youths from less-resourced neighborhoods responded with less anger (*p* < .001, marginal R^2^ = 0.42) and more sadness (*p* < .001, marginal *R*^2^ = 0.46) to the negatively biased condition than youths from well-resourced neighborhoods. On the neurobiological level, lower COI scores were associated with a more localized processing mode (*p*= .039, marginal *R*^2^= 0.076), reduced connectivity between the somatic–motor–salience and the control network (*p = .041,* marginal. *R*^2^= 0.040), and fewer provincial hubs in the somatic–motor–salience, control, and default mode networks (all *P*_FWE_ < .05

**Conclusion:**

The present study adds to a growing literature documenting how inequity may affect the brain and emotions in youths. Future work should test whether findings generalize to more diverse samples and should explore effects on neurodevelopmental trajectories and emerging mood disorders during adolescence.

**Plain language summary:**

A growing body of literature suggests that access to resources at the neighborhood level affects the neurodevelopment and mental health of youth. This study explores how access to neighborhood resources shapes the behavioral and neurobiological responses to negatively biased environments in youth. During brain imaging, 211 youth participated in a task where rewards were randomly withheld under the pretense that the youth performed poorly, an “unfair” intervention that elicits frustration. The authors found that youth from less-resourced neighborhoods exhibited less anger and more sadness in response to the unfair condition compared to youth from well-resourced neighborhoods. Limited access to neighborhood resources was also associated with reduced connectivity between the control and motor brain networks. These findings suggest that neighborhood inequity may impact the neurodevelopment and mental health of youth.

**Diversity & Inclusion Statement:**

One or more of the authors of this paper self-identifies as a member of one or more historically underrepresented racial and/or ethnic groups in science. One or more of the authors of this paper self-identifies as a member of one or more historically underrepresented sexual and/or gender groups in science. One or more of the authors of this paper received support from a program designed to increase minority representation in science. We actively worked to promote sex and gender balance in our author group. We actively worked to promote inclusion of historically underrepresented racial and/or ethnic groups in science in our author group.

A growing body of literature suggests that, during the formative stages of childhood and adolescence, access to resources at the neighborhood level (ie, quality of education and health care, exposure to crime rates, pollution) affects neurodevelopment^1–3^ and mental health.^4^ The precise mechanisms underlying these associations and dose–response relationships are poorly understood. Nevertheless, a prevailing hypothesis posits that neighborhood-based adversities amplify the probability of encountering stress-inducing circumstances.^5^ These circumstances may entail restricted access to resources and rewards. During the developmentally sensitive period of childhood and adolescence,^6^ the repetitive or protracted encounter of scenarios involving thwarted goal attainment or reward omission might shape neurodevelopmental trajectories of the underlying neural circuitry, consequently altering the affective and behavioral responses to such experiences. Here, we test this hypothesis in a pooled sample of 211 youths from 2 previously published studies^7,8^ examining whether and how even minor variations in the dimensional variable of access to resources (eg, quality of education and schools, safe housing, access to healthy food) influence affective, behavioral, and neurobiological responses to reward omission in an experimental task.

To date, work investigating the relationship between social determinants of health, neurodevelopment, and mental health has focused primarily on socio-economic resources at the household level.^9,10^ However, household- and neighborhood-level resources are not synonymous,^11^ and may affect mental health outcomes through independent pathways.^5^ Economic neighborhood characteristics have been linked to alterations in the functional connectivity^1^ and topology^3^ of intrinsic brain networks during resting state. Furthermore, using tasks to probe specific circuits, neighborhood disadvantage was associated with increased sensitivity of the amygdala to threat^2^ and increased responsivity of the reward circuit during the anticipation of rewards.^12,13^ However, our understanding of how neighborhood-level resources affect the developing brain may be enhanced by the use of novel metrics of neighborhood attributes, which, in addition to economic factors, incorporate contemporary youth-relevant neighborhood features in the areas of education, health, and environment, such as the Child Opportunity Index (COI).^14,15^

On an individual level, living in neighborhoods that limit access to resources might increase the frequency of situations in which one’s actions do not yield an anticipated reward. For example, a student might receive poor results in a state-wide test despite studying hard because their school could not provide appropriate learning resources. In animals, the omission of an expected reward is followed by a short-term increase in behaviors aimed at obtaining the blocked reward.^16^ In humans, the affective response to reward omission also includes increased frustration and sadness.^7,8^ On a neurobiological level, omitting an expected reward, in the literature referred toas frustrative non-reward, a construct closely related to negative prediction error,^17^ involves increased activity of widely distributed cortical and subcortical brain regions.^7,8,18^ How access to resources at the neighborhood level affects the multimodal response to blocked goal attainment in youths, that is, whether it leads to habituation (ie, reduced reactivity) or sensitization (ie, increased reactivity) and whether such effects also emerge in response to relatively small variations in access to resources are currently unknown.

Given repeated reports of broad effects of neighborhood attributes and widespread neurobiological responses to blocked goal attainment, graph-theoretical approaches appear particularly suited to study these phenomena. This framework assumes that brain regions (ie, nodes) form more or less delineated subnetworks (ie, modules). Recent work linked the omission of expected rewards to changes in brain network segregation (ie, how well the brain can be divided into non-overlapping modules), changes in the nodal composition of functional modules, and the efficiency of parallel information processing in the visual, default-mode, and frontal–temporal–limbic modules.^8^

Our primary goal was to test whether even small variations in access to neighborhood resources, a continuous variable likely to influence the frequency of experiences involving reward omission, affect the following: (1) self-reported frustration and sadness, (2) reaction time, and (3) brain network topology. Our secondary questions included whether observed effects were driven by neighborhood attributes in the economic, health, and/or education domain and whether findings were specific to neighborhood-level attributes or were equally related to household-level economic resources. We also examined the robustness of the effect by adding natal sex, age, psychotropic medication, or psychopathology as explanatory variables. Concerning psychopathology, we focused on irritability, a common symptom in youths that was previously linked to perturbed responses to reward omission.^7,8^

## METHOD

### Participants

Participants were initially recruited in 2 published studies^7,8^ designed to characterize the neurobiology of irritability. Here, we reanalyzed data from a pooled subset of these participants who had completed the COI measure (n/% of original samples: 168/86^7^, 48/72^8^). Compared to the previous studies, here we focus on different aspects of the task and use a different analytic approach for the imaging data. Our sample consisted of 211 youths (aged 8–18 years, mean age [SD] = 13.0 [2.4], 48% identifying as boys). Of the participants, 11% identified as African American, 3% as Asian, 62% as White, and 19% reported multiple racial identities. Twelve percent of participants indicated Hispanic or Latinx heritage. The sample was relatively affluent; the annual household income was >$180,000 for 23%, $90,000 to $180,000 for 53%, and <$90,000 for 21% of the sample. Based on household income and number of family members, 4% of the sample was below the poverty line. Thus, although our work does not speak to the effects of neighborhood resources in marginalized communities, it does shed light on the sensitivity of systems mediating responses to negatively biased environments to relatively small variations in such resources.

A master’s- or doctoral-level clinician administered the Kiddie Schedule for Affective Disorders (KSADS).^19^ Youths were included if they met the criteria for no psychiatric disorder (n = 72) or at least 1 of 3 disorders: anxiety disorder (n = 47), attention-deficit/hyperactivity disorder (ADHD; n = 55), and disruptive mood dysregulation disorder (DMDD; n = 56). Of the youths, 27% were taking psychotropic medication, including stimulants (n = 49), non-stimulant ADHD medication (n = 11), antidepressants (n = 27), antipsychotics (n = 7), and anticonvulsants (n = 10). Participants scored an average of 112 (SD = 12) on the Wechsler Abbreviated Scale of Intelligence.^20^ Exclusion criteria were neurological disorders, autism spectrum disorders, psychosis, bipolar disorders, posttraumatic stress disorder, substance use, magnetic resonance imaging (MRI) contraindications, pregnancy, and Full Scale IQ <70. We obtained institutional review board approval, parent or guardian consent, and child assent.

### Symptom Ratings

We used the Screen for Child Anxiety-Related Emotional Disorders (SCARED)^21^ to measure anxiety, the Comprehensive Behavior Ratings Scale (CBRS)^22^ to quantify inattention and hyperactivity, the Affective Reactivity Index (ARI)^23^ to evaluate irritability, and the Children’s Depression Inventory (CDI)^24^ to assess depressive symptoms. Descriptive information regarding these scales is available in Table S1, available online.

### Neighborhood Resources

**FIGURE 1:**
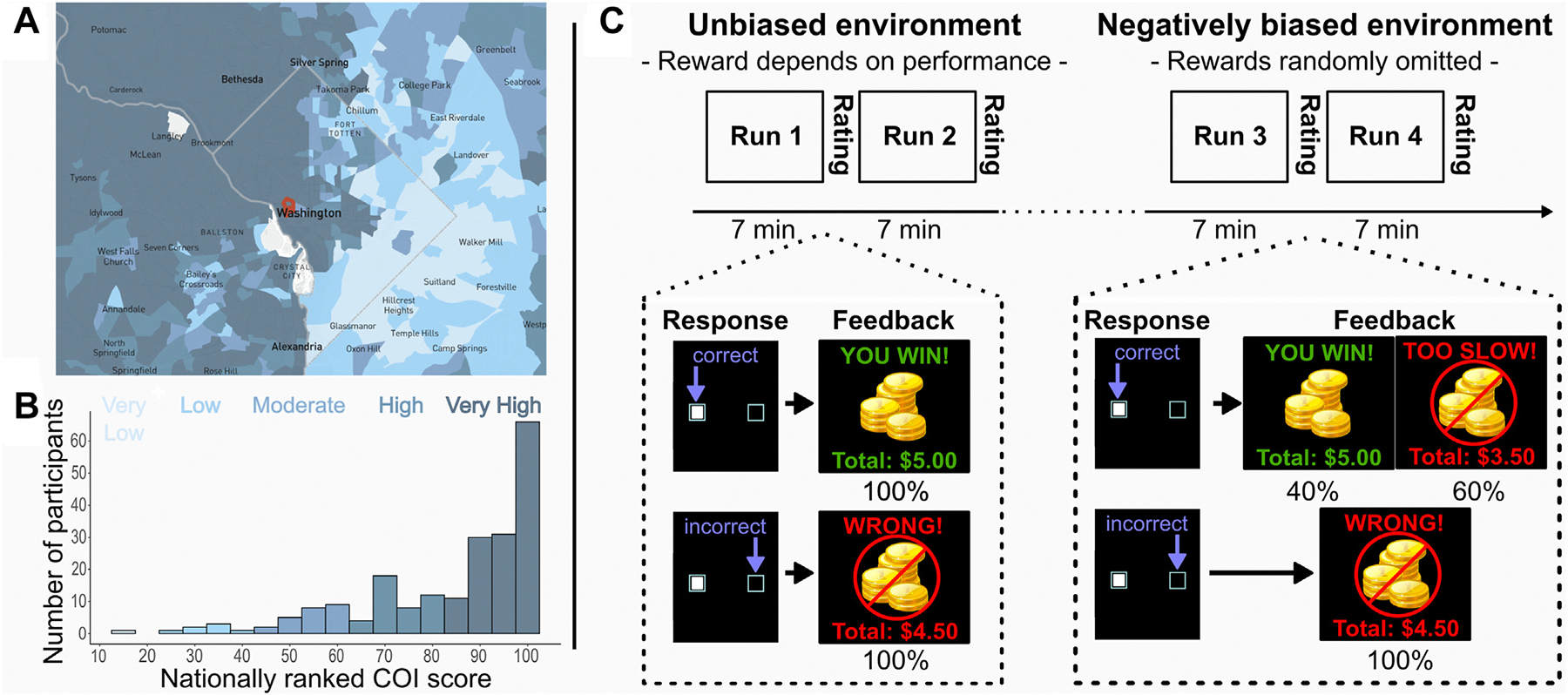
Illustration of the Independent Variables Used in This Study **Note:** Panel A displays the nationally ranked Childhood Opportunity Index (COI) overlaid on the Washington, DC, area, where most participants were living.^25^ Panel B shows the distribution of the COI score in our sample. Panel C shows the paradigm modeling unbiased and negatively biased environments.

We measured neighborhood resources with COI 2.0,^14,15^ which allowed us to compare the level of opportunity that neighborhoods provide for children across the United States in a single metric. The COI 2.0 is a composite of 29 indicators that measure neighborhood-based opportunities for youths in 3 domains: education (eg, early childhood education enrollment, third-grade math/reading proficiency, adult educational attainment); health and environment (eg, access to healthy food, health insurance coverage, walkability); and social and economic opportunities (eg, poverty rate, public assistance rate, single-headed households, home ownership, high-skill employment). The tool evaluates these risks and resources in geographic regions defined by the United States Census Bureau (ie, neighborhoods of approximately 4,000 people). It is a relative measure that indicates the level of opportunity in a neighborhood relative to the other >72,000 defined neighborhoods in the United States. In our analyses, we used the COI rank score, which ranges from 0 (lowest resource level) to 100 (highest resource level; for a distribution of the COI in our sample, see Figure 1A^25^ and B).

Information on how the COI rank score is distributed across racial, ethnic, and diagnostic categories is provided in Figures S1 to S3, available online. It is important to note that the COI score and racial/ ethnic categories are confounded, such that COI scores tend to be lower for Black and Hispanic children.^14^ However, it is the unequal neighborhood-based opportunity that is thought to lead to inequities in outcomes (eg, neurodevelopment and mental health) by race and ethnicity, not these social constructs themselves. For that reason, race and ethnicity (ie, correlates of neighborhood opportunity) were not included as covariates in our regression models.

### Paradigm

All participants completed the Affective Posner Task,^7,8^ which requires youths to indicate whether a target appeared on the left or right side of the screen. The target was preceded by a cue correctly stating the target location in 75% of trials. In the remaining trials, the cue was an invalid predictor of the target location, and participants had to reorient their attention. The task was easy; participants responded correctly in 96% of the first 100 trials and received $0.50 for each correct response. During these first 100 trials (divided into 2 runs), the participant’s performance determined the monetary reward. So, these trials are viewed as a model of an unbiased and fair environment. However, during the second 100 trials, the feedback was rigged without the participants’ knowledge, modeling reward omission in a negatively biased, unfair environment. More specifically, in 60% of correct trials, youths received feedback that they responded too slowly, and $0.50 was deducted for each trial (Figure 1C). The order of the 2 conditions was the same across participants, as the negatively biased environment induces feelings of frustration and sadness, which might alter neural network configuration for up to 10 minutes.^8^ We obtained frustration and sadness ratings after each of the 4 runs on a 9point Likert scale.

Previous reports^7,8^ refer to the 2 conditions as reward vs frustrating conditions. However, given the focus on environments in this study, we will refer to them as the unbiased vs negatively biased condition; we suggest that the latter models aspects of low-opportunity neighborhoods. Given our interest in the environment, we analyzed the imaging data as blocks, which is different from the original reports that focused on task events^7,8^ and the recovery of neural systems after task.^8^

### Imaging Data

#### Data Acquisition.

Imaging data were acquired on two 3.0 Tesla General Electric Discovery MR750 scanners with an 8-channel^7^ or 32-channel^8^ head coil. Blood-oxygen-level–dependent (BOLD) changes during the task were measured with a T2*-weighted sequence (Tseng *et al*.^7^/ Linke *et al*.^8^: TR = 2300 msec, TE = 25/ 30 milliseconds, flip angle 75° [n = 113] or 90° [n = 55] / 70°, field of view = 240 mm^2^, matrix size = 96×96, 41/44 axial interleaved slices, slice thickness 3 mm, 206/179 volumes per run). We also acquired a high-resolution T1-weighted image with a 3D standard sequence (Tseng *et al*.^7^/ Linke *et al*.^8^: TE = min, TI = 725/425, field of view = 256×256×124/256×256×176 mm, matrix size = 298×298×103/256×256×176). Sequence and scanner were added as nuisance variables in the imaging analyses.

In the negatively biased condition, imaging data was available for all participants, but in the unbiased condition, imaging data was available from only 80% of the participants. This was because, in 1 study,^8^ the unbiased condition was completed outside the scanner. Thus, we focused our imaging analysis on the negatively biased condition but explored the specificity of our findings to the unbiased condition in the subsample with imaging data.

#### Data Preprocessing.

Data were preprocessed with FMRIPREP version 22.0.0.^26^ This pipeline created a reference image and mask based on the initial functional volumes and then performed a series of spatial transformations in 1 step, including slice time correction using 3dTshift from AFNI, motion correction using MCFLIRT from FSL,^27^ and co-registration to the structural volume using boundary-based registration with 9 degrees of freedom.^28^ ICA-based Automatic Removal of Motion Artifacts (AROMA) was used to non-aggressively denoise the time courses.^29^ Structural scans were subjected to FreeSurfer processing.

#### Brain Networks.

We extracted the time course from 100 cortical parcels previously assigned to known intrinsic functional networks^30^ and 16 subcortical regions from the FreeSurfer segmentation (ie, bilateral nucleus accumbens, caudate, pallidum, putamen, amygdala, hippocampus, thalamus, and ventral diencephalon). We regressed the white matter and cerebrospinal fluid signals, and the ICAAROMA components classified as head motion from the time series. We also regressed the onset of the cue, response, and feedback. We quantified functional connectivity as Pearson correlations. All correlation coefficients underwent Fisher z^0^-transformation to ensure normality. The preprocessing resulted in two 116 × 116 connectivity matrices per participant: 1 matrix for the unbiased and 1 matrix for the negatively biased condition.

### Graph Analysis

**FIGURE 2:**
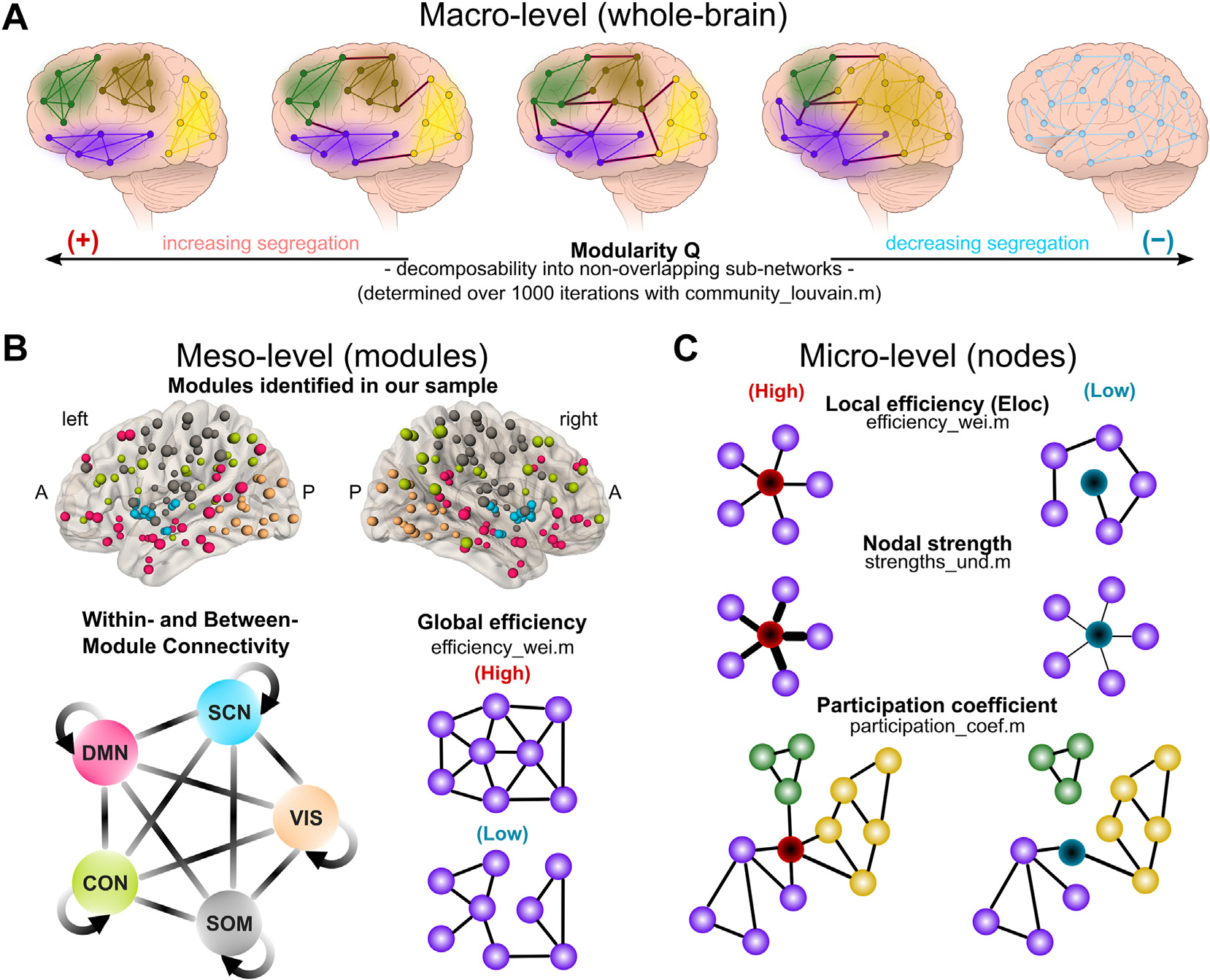
Schematic of Key Graph-Theoretic Metrics at the Macro- (Whole Brain), Meso- (Modules), and Micro- (Nodes) Levels Used in This Study **Note:** Panel A illustrates how high and low modularity values correspond to brain network topology. Panel B shows the coordinates of the center of the parcels from which time courses were extracted as circles over the ICBM152 brain template. The colors of the circles signify the 5 modules identified in our data. More specifically, apricot equals the visual network (VIS), gray indicates the somatic–motor–salience network (SOM), green symbolizes the control network (CON), pink signifies the default mode network (DMN), and light blue stands for the subcortical network (SCN). The lower part of Panel B illustrates within- and between-module connectivity and differences between high and low intramodular global efficiency. Panel C illustrates high and low values of the 3 micro-level metrics. For the metrics derived from the brain connectivity toolbox, the figure also indicates the script from the brain connectivity toolbox that was used to calculate the respective metric. COI = Childhood Opportunity Index.

We analyzed the relationship between the COI score and neural network topology in the negatively biased condition using key metrics obtained from the brain connectivity toolbox^31^ using weighted undirected brain networks as input. Our analyses focused on 3 levels of scale: (1) the brain as 1 big network at the macro level (Figure 2A); (2) functional subnetworks of the brain (ie, modules, Figure 2B) at the meso-level; and (3) features of single parcels (ie, brain regions or nodes, Figure 2C) at the local level.

Based on previous work associating reward omission with changes in brain network segregation,^8^ whole-brain analyses focused on modularity (Q), which describes how well the brain can be divided into non-overlapping modules. Higher Q values indicate more localized, segregated information processing. Q values were estimated using the Louvain greedy algorithm.^32^ Given the stochastic initialization of the greedy optimization, the algorithm was applied 1,000 times, and the highest Q value (Q_max_) was used for analyses.

Next, we investigated the functional modules in more depth. We used sample-specific modules identified in a multistep procedure over the pre-defined networks from the Schaefer atlas because the adult brain topology represented by the Schaefer parcellation emerges only during childhood and adolescence^33^ (Figure S4, available online). First, we used a consensus approach to calculate an agreement matrix across the 1,000 iterations that identified Q_max_ for each participant. This matrix, which codes the tendency for each pair of nodes to be assigned to the same module across iterations, served as input for an independent module partitioning. From this, we calculated a matrix reflecting the probability of nodes being assigned to the same module across all 211 participants.^8^ This matrix was then subjected to the Louvain greedy algorithm (see above) to detect sample-specific modules. Consistent with prior work,^8^ we estimated within-module global efficiency (E_glob_), quantifying the module’s capacity for parallel information processing. However, as prior findings^8^ between frustration increase and E_glob_ were limited to the receipt of monetary reward in the negatively biased condition, we additionally calculated the average within- and between module functional connectivity to test for module-level associations with the COI.

Finally, based on work reporting associations between neighborhood attributes and provincial hubness,^3^ we calculated 3 metrics of connectedness at the nodal level: local efficiency (E_loc_), nodal strength, and the participation coefficient. E_loc_ shows how well nodes are connected with their immediate neighboring nodes, whereas nodal strength quantifies how well connected single nodes are to all other nodes in the same module.^34^ Both measures are indicators of provincial hubness. Finally, the participation coefficient indicates how well connected a node is to nodes from other modules (ie, its relevance of a node for intermodular communication^35^).

### Data Analysis

We used linear mixed-effects models (R: lme4 package) to analyze task-induced feelings (ie, frustration and sadness), task behavior (ie, reaction time and accuracy), and neurobiological responses at the whole-brain and modular levels. For the task-induced feelings and task behavior, the baseline model contained condition (unbiased vs negatively biased) and primary study^7,8^ as fixed effects and had random slopes for the participants. In our main model, we added the continuous COI scores and COI score condition as fixed effects and random slopes for the 22 micro areas where participants lived. For whole-brain and module-level analyses, the baseline model contained the original studies,^7,8^ sequence, scanner, and mean framewise displacement as fixed effects. In the primary model, the COI score and random slopes for the micro areas were added. Significance was determined through likelihood ratio tests of the model with the COI score against the baseline model. We report marginal *R*^2^ representing the variance explained by the fixed effects and conditional *R*^2^, interpreted as the variance explained by the full model.^36^ Residual plots were visually inspected to ensure that there were no apparent deviations from homoscedasticity or normality.

The relationship between the COI score and nodal-level graph metrics was determined using 5,000 permutations and a threshold of *p* < .05 corrected for family-wise error rate (FWER) across nodes and contrasts. Analyses included original study, scanner, sequence, and mean framewise displacement as nuisance variables. We accounted for the nested nature of the data through exchangeability blocks.

For models yielding significant results, we performed secondary analyses testing whether the effects were driven by specific COI domains (ie, education, health and environment, and social and economic opportunities) and whether the effects could be observed when using socioeconomic status (A.B. Hollingshead, 1975; unpublished), an indicator of household-level resources, as the independent variable. We also confirmed that effects were not explained by age, natal sex, current symptomatology (ie, ratings of irritability, anxiety, and depressive symptoms), or psychotropic medication load,^37^ as there was no washout period prior to scanning by adding the respective variables and the interaction term of these variables with COI as fixed effects. We also explored the direct effects of the COI score on ratings of irritability, anxiety, and depressive symptoms.

## RESULTS

### Neighborhood Opportunity and Reward Omission

#### Affective Response.

**FIGURE 3:**
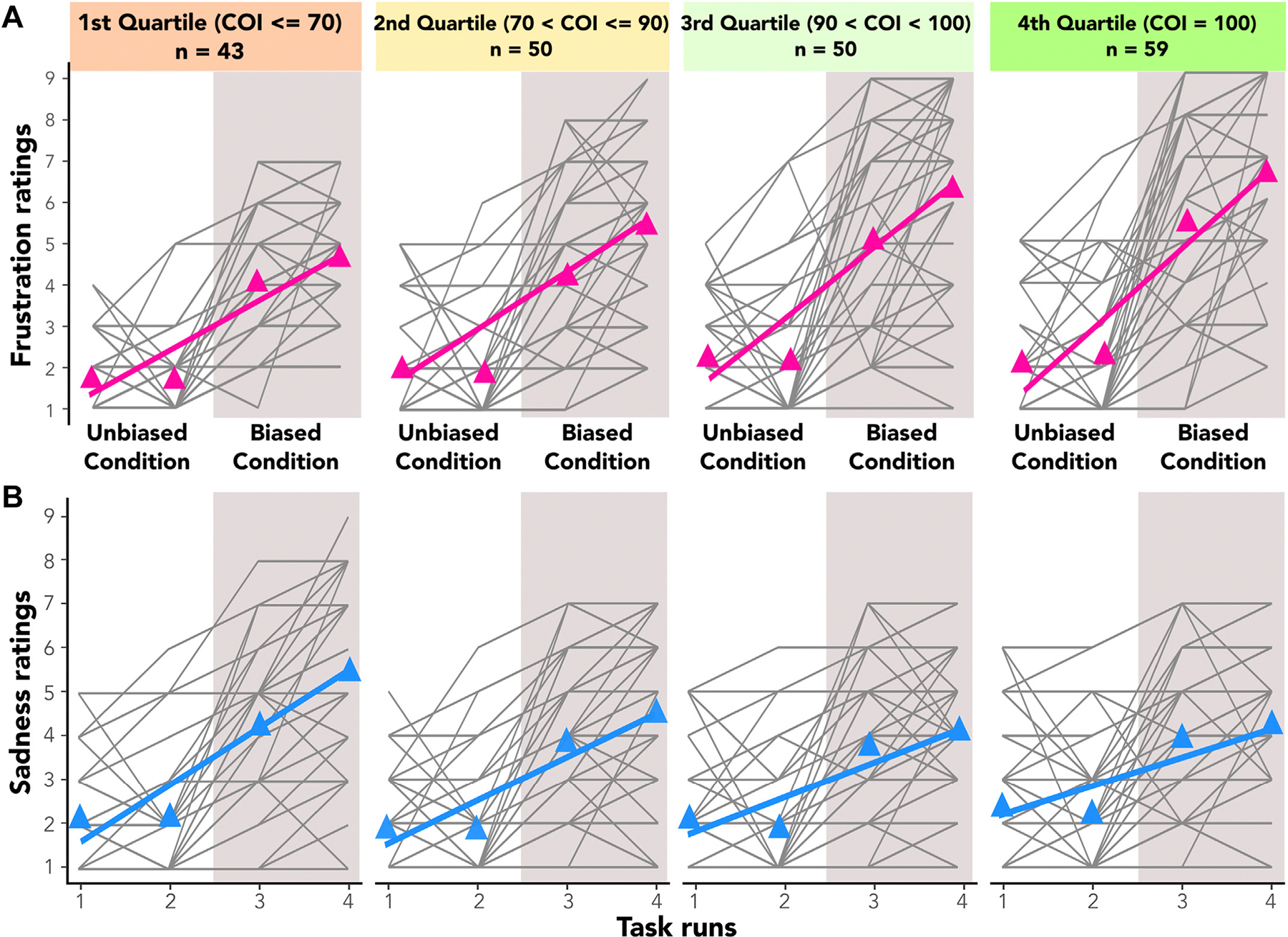
Effect of Neighborhood Opportunity on Frustration (Panel A) and Sadness (Panel B) **Note:** Gray lines represent the raw data, and colored lines show the median effect of the respective Childhood Opportunity Index (COI) quartile specific to our sample shown at the top in the traffic-light color scheme.

Across subjects, exposure to our model of a negatively biased environment (ie, the condition in which rewards were not determined by performance but were randomly omitted in 60% of the trials) was associated with an increase in frustration (*β* = 3.2) and sadness (*β* = 1.9). The models containing the condition × COI interaction term fit the data better than the baseline model (frustration: *β* = 0.02, χ^2^_(3)_ = 18.61, *p* < .001, marginal *R*^2^ = 0.42, conditional *R*^2^ = 0.66 [Tables S2 and S3, available online]; sadness: *β*=0.02, χ^2^_(3)_ = 32.06, *p* < .001, marginal *R*^2^ = 0.46, conditional *R*^2^ = 0.62 [Tables S4 and S5, available online]). To interpret this interaction between the continuous COI score and the 2 task conditions (ie, unbiased vs biased environment), we divided the sample by COI and plotted the raw data and the mean response of these subsamples (frustration: Figures 3A and S5A, available online, sadness: Figures 3B and S5B, available online). These graphs show that youths living in neighborhoods with relatively lower COI scores displayed a flatter increase in frustration, but a steeper increase in sadness from the unbiased to the biased condition relative to youths living in well-resourced neighborhoods.

#### Behavioral Response.

The negatively biased condition was associated with faster responses (*β* = −208). This was expected, as rewards were withheld under the pretense that youths did not respond fast enough. However, this effect was more pronounced when the cue was a valid predictor of the target location (condition × validity: *β*= 43.1). Adding COI score did not improve the model fit (χ^2^_(5)_ = 6.84, *p* = .232). The COI score had no effect on accuracy (χ^2^_(5)_ = 4.95, *p* = .421).

#### Neurobiological Response.

At the whole-brain level, lower COI scores were associated with higher Q values (*β* = −0.0003, χ^2^_(1)_ = 4.22, *p* = .039, marginal *R*^2^ = 0.076 [Tables S6 and S7, available online]), which indicate a more segregated brain network topology and more localized information processing.

Subsequent analyses focused on the 5 modules identified in our sample during the negatively biased condition (ie, visual [VIS], somatic–motor–salience [SOM], control [CON], default mode [DMN], and subcortical [SCN] networks [Figure 3B and S2, available online]). At this modular level, lower COI scores were associated with lower FC between the CON and SOM (*β*= 0.0003, χ^2^_(1)_ = 4.17, *p* = .041, marginal *R*^2^= 0.040, conditional *R*^2^= 0.400 [Tables S8 and S9, available online]). There were no findings using within-network FC (all *p* > .055) or E_glob_ (all *p* > .123).

Finally, lower COI scores were linked to reduced within-module connectedness at the nodal level. Specifically, we saw reduced E_loc_ in 22 nodes, the majority of which were located in the SOM (n = 6), CON (n = 5), or DMN (n = 7, *p*_FWER_ < .05) (Figure 5). Four DMN nodes and 1 SOM node also displayed lower nodal strength (*p*_FWER_ < .05). Lower COI scores were also associated with a lower participation coefficient of 1 node in the left temporal lobe belonging to the DMN (*p*_FWER_ < .05).

**FIGURE 4:**
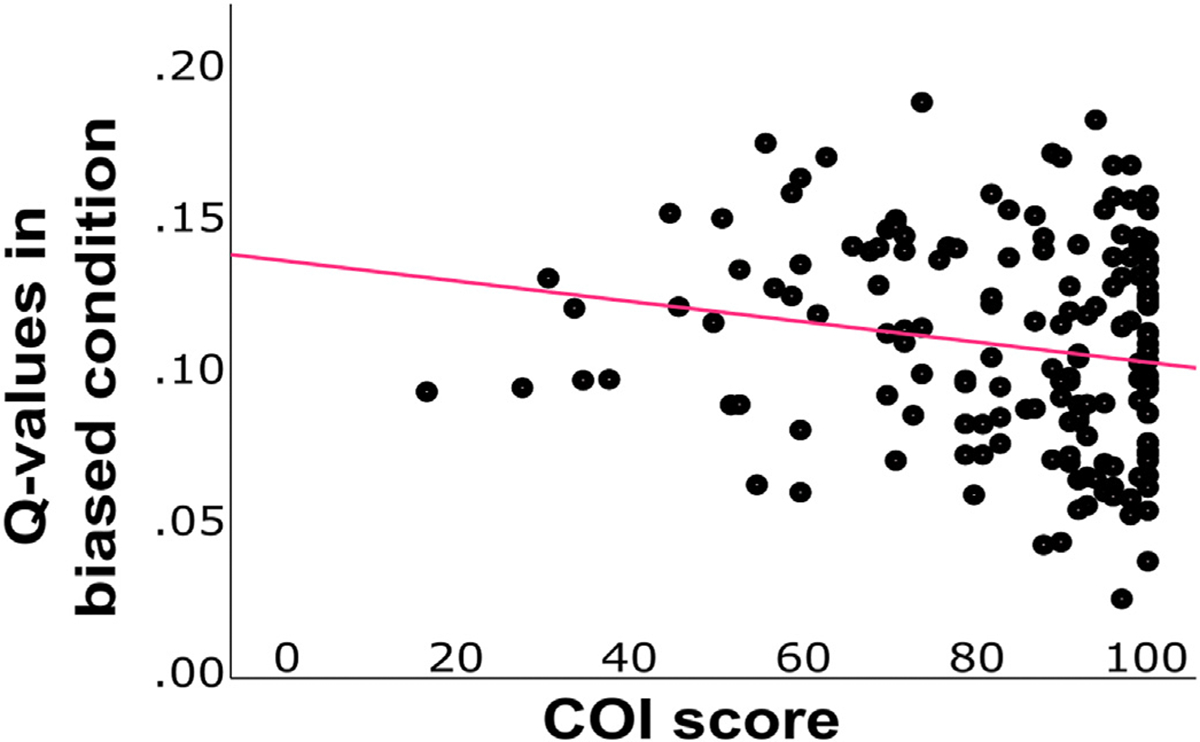
Effect of Neighborhood Opportunity on Brain Network Segregation Operationalized Through Q Values **Note:** The upper panel shows that youths from lower-opportunity neighborhoods show higher Q-values (ie, a more localized information processing). The lower panel, in which the difference in Q between the biased and unbiased conditions is shown on the y-axis, supports the notion that this relationship between Childhood Opportunity Index (COI) score and Q values emerges during the biased condition.

Specificity analyses in the subsample with imaging data during the unbiased condition suggest that the described neurobiological signature partially emerged during exposure to the negatively biased environment. Specifically, we saw a COI score task condition interaction for modularity (*β* = −0.0003, χ^2^_(2)_ = 7.79, *p* = .020, marginal *R*^2^ = 0.232, conditional *R*^2^ = 0.232 [Figure 4]), and E_loc_ of the nodes in the DMN and CON, where we saw a COI effect in the primary analysis (all *p* < .0431).

### Secondary Analyses

#### COI Domain Subscores.

The frustration and sadness effects were present when using each of the 3 COI domain scores (all *p* < .035 [Tables S2 and S4, available online]). Effects of COI domain on the neurobiological level were inconsistent (Tables S6 and S7, available online).

#### Household-Level Resources.

Using the family’s socioeconomic status instead of the COI score yielded no significant results (all *p* > .125).

#### Covariates.

All effects of the COI score remained significant when accounting for age, natal sex, medication load, psychopathology (ie, parent- and youth-rated ARI or SCARED scores, or youth-rated CDI scores) or the respective interaction terms (eg, COI score × parent-rated ARI). There were several effects of these covariates across the sample. Specifically, parent- and youth-rated ARI related to a steeper increase in frustration (parent-rated ARI: *β*= 0.06, youth-rated ARI: β = 0.11) and sadness (parent-rated ARI-P: *β* = 0.06, youth-rated ARI: *β*= 0.11). Parent-rated ARI was also negatively associated with Q (*β* = −0.001, χ^2^_(1)_ = 4.16, *p* = .041) and positively related to the participation coefficient of 4 nodes in the VIS, SOM, and CON. Our data did not support a COI × parent-rated ARI interaction (all *p*_FWER_ > .05). Results remained when psychiatric disorders instead of symptoms were used as covariates; there were no direct effects of diagnostic categories (all *p* > .22).

#### Direct Effects of COI on Psychopathology.

Exploratory analyses regarding the relationship between the COI score and parent- or youth-reported irritability, parent- or youth-reported anxiety, or youth-reported depressive symptoms (for descriptive statistics, see Table S1, available online) yielded null findings (all *p* > .372).

## DISCUSSION

**FIGURE 5:**
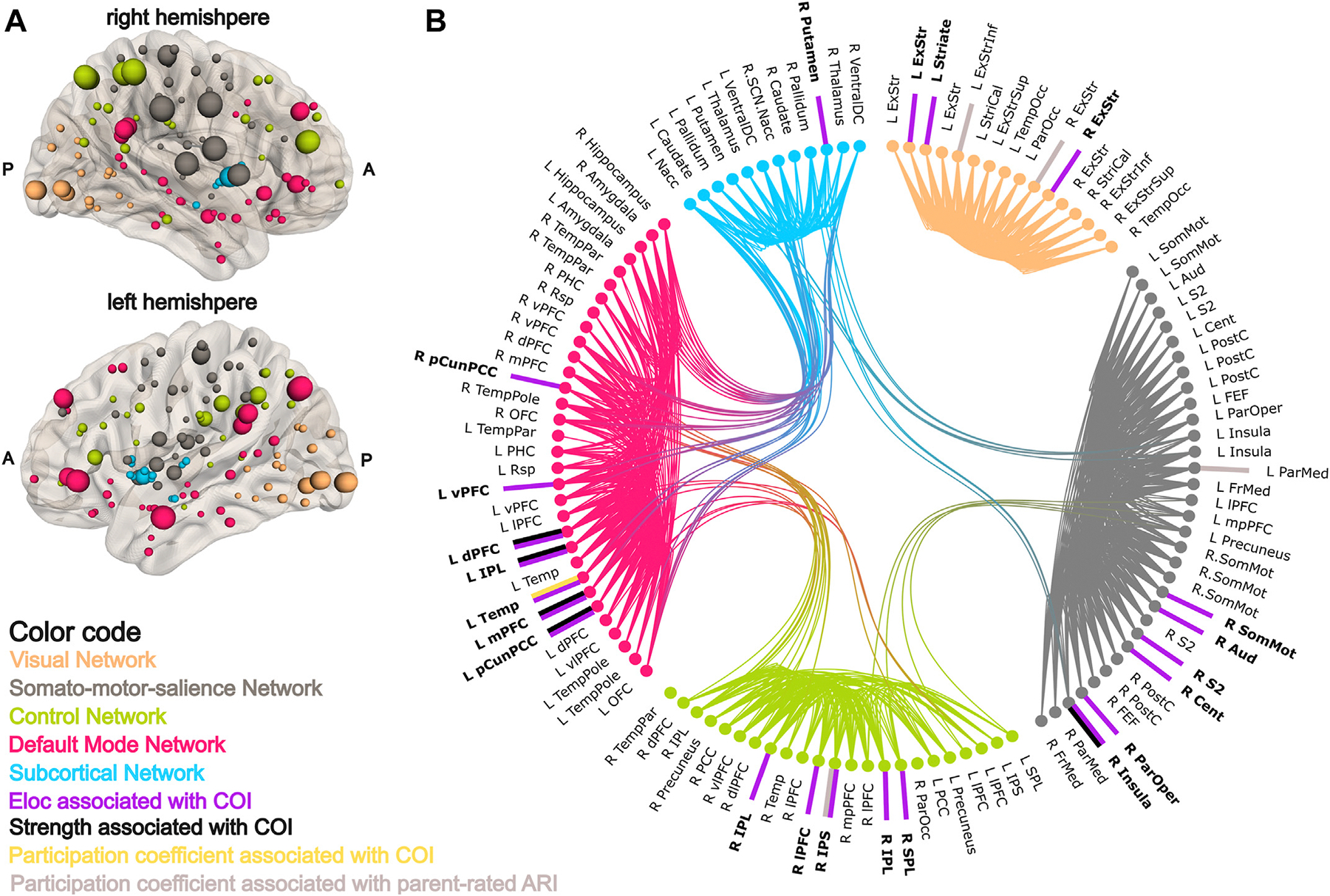
Effect of Neighborhood Resources and Parent-Rated Irritability on Provincial Hubness, a Property of Single Nodes **Note:** Panel A shows the coordinates of the center of the parcels from which time courses were extracted as circles over the ICBM152 brain template. Larger circles indicate the nodes where we saw lower Eloc in youths from low-opportunity neighborhoods. Panel B shows all regions belonging to a module and the edges identifiable across participants. The presence and color of the bars emerging from the circles representing the brain regions indicate whether youths from lower-opportunity neighborhoods showed lower Eloc (purple), strength (black), or participation (gold) for this node, and whether there was a relationship between parent-rated irritability and participation (gray). A = anterior; dPFC = dorsal prefrontal cortex; Fef = frontal eye field; FrMed = medial frontal cortex; IPL = inferior parietal lobe; IPS = intraparietal sulcus; L = left hemisphere; mPFC = medial prefrontal cortex; Nacc = nucleus accumbens; OFC = orbitofrontal cortex; P = posterior; ParMed = medial parietal cortex; ParOper = parietal operculum; PCC = posterior cingulate cortex; PFC = prefrontal cortex; PHC = parahippocampal cortex; R = right hemisphere; SomMot = somatomotor cortex; SPL = superior parietal lobe; Temp = temporal; TempPar = temporoparietal region; TempPole = temporal pole; ventral DC = ventral diencephalon (which includes hypothalamus, mammillary bodies, subthalamic nuclei, substantia nigra, red nucleus, and medial and lateral geniculate nuclei); vPFC = ventromedial prefrontal cortex; vlPFC =ventrolateral prefrontal cortex.

During late childhood and adolescence, youths are increasingly exposed to determinants of health that reside within neighborhoods. Our data indicate that the availability of youth-relevant community resources may shape individual responses to negatively biased environments that block desirable rewards. Specifically, our findings suggest that, following the omission of an expected reward, access to resources is positively associated with an increase in frustration but negatively associated with an increase in sadness. On a neurobiological level, youths from neighborhoods with fewer resources showed a more localized processing mode brain-wide during the negatively biased condition, as well as reduced FC between the SOM and CON, which largely consisted of prefrontal and parietal brain regions (Figure 5), and reduced provincial hubness of 22 regions primarily located in the SOM, CON, and DMN. These effects were robust when accounting for sociodemographic and clinical variables, and seemed specific to neighborhood-compared to household-level resources. This study extends the growing literature linking neighborhood attributes to neurodevelopment and mental health by shedding light on potential mechanisms.

Exposure to disadvantaged neighborhoods has been shown to elicit greater negative affective and physiological stress responses in experimental settings.^38^ Associations between neighborhood characteristics, emotional problems (eg, anxiety and depressive symptoms^39,40^), and perturbations in the physiological stress response^5^ suggest that chronic exposure to disadvantaged environments leads to adaptations in these immediate reactions with long-term mental health consequences. The present study extends our mechanistic understanding of this process.

Consistent with previous reports that frequent exposure to threats at the neighborhood level may lead to habituation or suppression of the fear response,^41,42^ youths living in neighborhoods with fewer resources responded with less anger to the omission of expected rewards. We propose that, compared to advantaged youths, those from less-resourced neighborhoods more frequently experience or observe that actions do not yield an intended outcome. This might affect their behavior-outcome expectancies, so the omission of a reward elicits a smaller negative prediction error and thus less frustration. In contrast, youths from well-resourced neighborhoods would expect their behaviors to lead to the expected outcome. So, the unexpected omission of a reward should elicit a larger negative prediction error and more intense frustration. Mechanistically, such learning, however, would be different from habituation, a form of non-associative learning whereby a response to a stimulus decreases after repeated or prolonged exposure, hypothesized to underlie alterations in the physiological stress response. These hypotheses could be tested in future work that would measure reward anticipation and model prediction errors.

Alterations in behavior–outcome expectancies could also explain why the predicted rise in sadness following goal obstruction was much steeper in youths living in areas with more restricted access to resources. In their seminal work, Seligman and Maier^43^ showed that when actions and outcomes are seemingly unrelated, a depressive state emerges. Here, we did not find previously reported associations between neighborhood features and depressive symptoms.^41,42^ However, the flattened increase in frustration and steeper increase in sadness might indicate the beginning of a process that could eventually lead to depressive symptoms, a hypothesis that needs testing.

So, would it be beneficial for youths from less-resourced neighborhoods to respond to negative bias with more anger and less sadness? More frustration and less sadness might be associated with greater efforts to obtain the blocked rewards. Such increased efforts could be related to increased motivation to work harder or attempts to change the system. Future work is needed to understand these complex relationships better.

On a neurobiological level, access to neighborhood resources was linked to adaptations at different levels of scale, ranging from brain network topology to regional hubness. Specifically, fewer opportunities at the neighborhood level were associated with a more segregated brain network topology (ie, higher modularity) in the negatively biased condition only. In previous work, increased brain network segregation has been shown to emerge after the omission of an expected reward while anticipating the outcome of the next attempt to obtain the reward and in the aftermath of the exposure to repeated goal obstruction.^8^ Transitions to a more localized processing mode are critical for motor execution^44^ and have been associated with faster responses in prior work using this task.^8^ However, a localized processing mode might be less beneficial for more cognitively demanding tasks^44^ and has also been linked to attention lapses during selective attention tasks.^45^ Furthermore, reduced connectivity between the CON and SOM might suggest diminished top-down control of behavior following reward prediction. In sum, our findings of increased modularity and weaker functional connectivity between the SOM and CON might represent an advantage when a motor response is required (including reacting quickly when faced with a negatively biased environment), but could be suboptimal in the context of more cognitively demanding tasks; this hypothesis could be tested in future work.

Prior work associated neighborhood disadvantage with broad reductions in brain connectivity,^46^ particularly in sensorimotor systems and higher-order networks such as CON and DMN. In our study, we saw positive associations between neighborhood opportunities and E_loc_ and nodal strength in 22 nodes within these networks during the exposure to the negatively biased condition. E_loc_ indicates dense connections between neighboring nodes so that information can be efficiently exchanged even if the node for which E_loc_ is estimated fails.^47^ Thus, fewer neighborhood resources might lower the error tolerance of the SOM, CON, and DMN, a vulnerability that appears to be triggered by a negatively biased environment. In youths from marginalized communities, this could be associated with perturbed maturation of brain networks,^3^ a hypothesis that could be tested in future longitudinal work.

The processing of emotional stimuli, including angry and sad faces, has been previously linked to changes in brain network segregation^48^ and hubness.^49^ Consistent with our findings, emotional hubs relevant to emotion perception and identification were located in the medial and lateral prefrontal areas, parietal regions, and insula.^49^ Both literature and our findings support the idea that neighborhoods shape the perception and identification of emotions, a hypothesis that needs further investigation.

Associations between neighborhood opportunities and frustration, sadness, and changes in brain network topology were independent of symptom levels. However, consistent with prior publications,^7,8^ irritability was related to the multimodal responses to reward omission. Here, we extend prior reports that linked irritability to higher activity of several brain regions, particularly in younger children,^7^ and altered efficiency of information in the aftermath of reward omission.^8^ We show that, at the nodal level, parent-rated irritability is positively linked to the participation coefficient of 2 VIS nodes and 1 SOM and CON node each. Thus, future work into the neurobiological underpinnings of irritability should encompass the study of regions relevant to the flow of information between these functional modules.

This study is based on cross-sectional data from a modestly sized sample of predominantly White and affluent participants. Although it is interesting that even relatively small variations in access to neighborhood resources are associated with differential emotional and neurobiological responses to reward omission, future work is needed to replicate these findings in samples with higher racial diversity and a substantial number of participants living in neighborhoods with low and very low COI scores. Furthermore, longitudinal studies examining associations between observed mood changes and depressive symptoms and developmental trajectories of brain network characteristics are warranted.

This study showed that, in youths, even mildly restricted access to resources at the neighborhood level is associated with altered responses to repeated reward omission in a negatively biased environment. Specifically, youths responded with less frustration but more sadness and more localized processing at the neurobiological level. The absence of associations between access to neighborhood resources and either symptomatology or changes in brain network configuration outside the negative bias condition highlights the need for future longitudinal work investigating dose–response relationships. Our results highlight the relevance of neighborhood conditions during development, and support neighborhood-level interventions to promote healthy development.

## Supplementary Material

supplement
