# Supplementary material for: Associations Between Neighborhood Resources and Youths’ Response to Reward Omission in a Task Modeling Negatively Biased Environments
Source: J Am Acad Child Adolesc Psychiatry. Author manuscript; Available in PMC 2026 Jun 26. (PMC13308576; doi:10.1016/j.jaac.2024.05.011)
Supplement: supplement [file NIHMS2183773-supplement-supplement.pdf]

Supplement 1

Associations between neighborhood resources and youth's response to reward omission in a task modeling negatively biased environments

Table S1. Descriptive statistics of core symptom dimensions of the included psychiatric diagnoses. All measures demonstrate acceptable to good retest-reliability (SCARED: ICC of parent/youth rating = .86/ .62, CBRS: r of parent-rating = .42, ARI: ICC of parent/youth rating = .85/ .78, CDI: r = .75).

	Mean	Median	SD	Min	Max	Skewness	Kurtosis
ARI-P	3.41	2	3.58	0	12	0.76	−0.7
ARI-Y	2.85	2	2.74	0	12	1.2	1.1
SCARED-P	15.6	12.5	13.14	0	61	0.94	0.39
SCARED-Y	18.11	15	13.45	0	62	1.03	0.75
CDI-Y	6.8	5	6.15	0	26	1.01	0.12
CBRS-ATT	58.57	54	14.62	43	90	0.7	−0.73
CBRS-HYP	58.77	56	13.38	40	90	0.49	−0.78

Note: ***ARI* =** Affective Reactivity Index measuring irritability; ***ATT* =** Attention; ***CBRS* =** Conners Behavioral Rating Scale measuring symptoms of Attention-Deficit/Hyperactivity Disorder; ***CDI* =** Child Depression Inventory; ***HYP* =** Hyperactivity; ***SCARED* =** Screen for Child Anxiety-Related Emotional Disorders; ***SD* =** standard deviation; ***-P* =** parent rating; ***-Y* =** youth rating

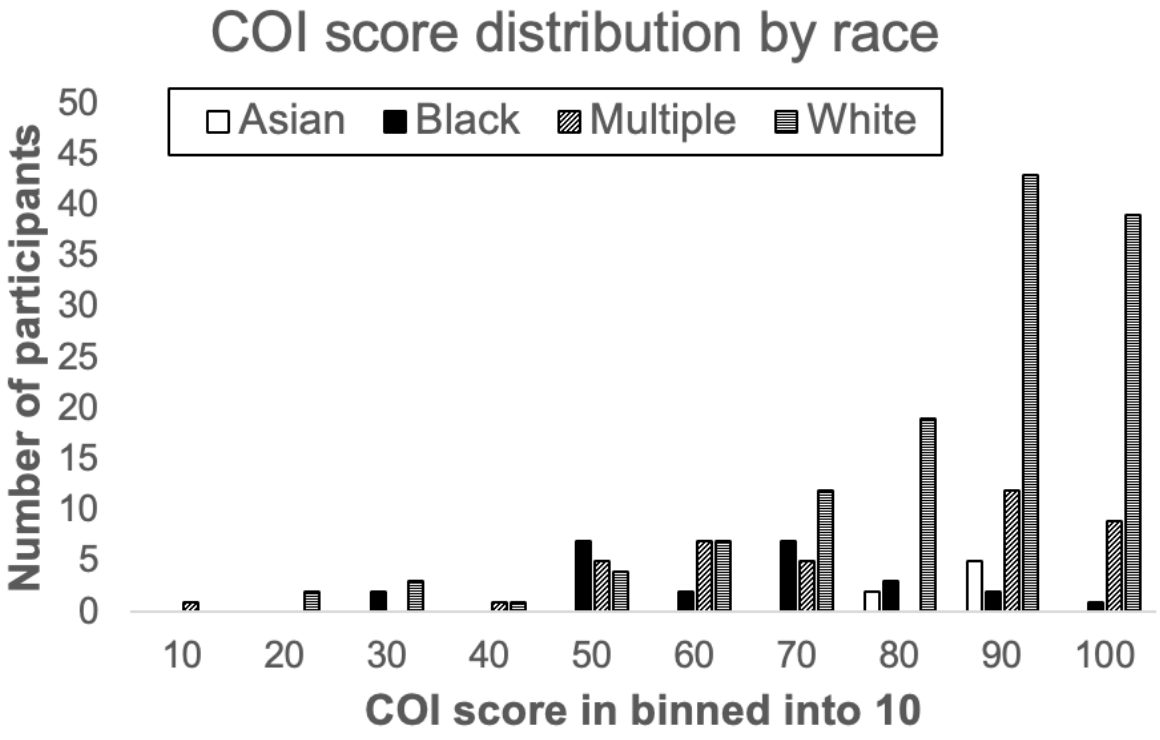

Figure S1. Distribution of the national ranked COI score by race

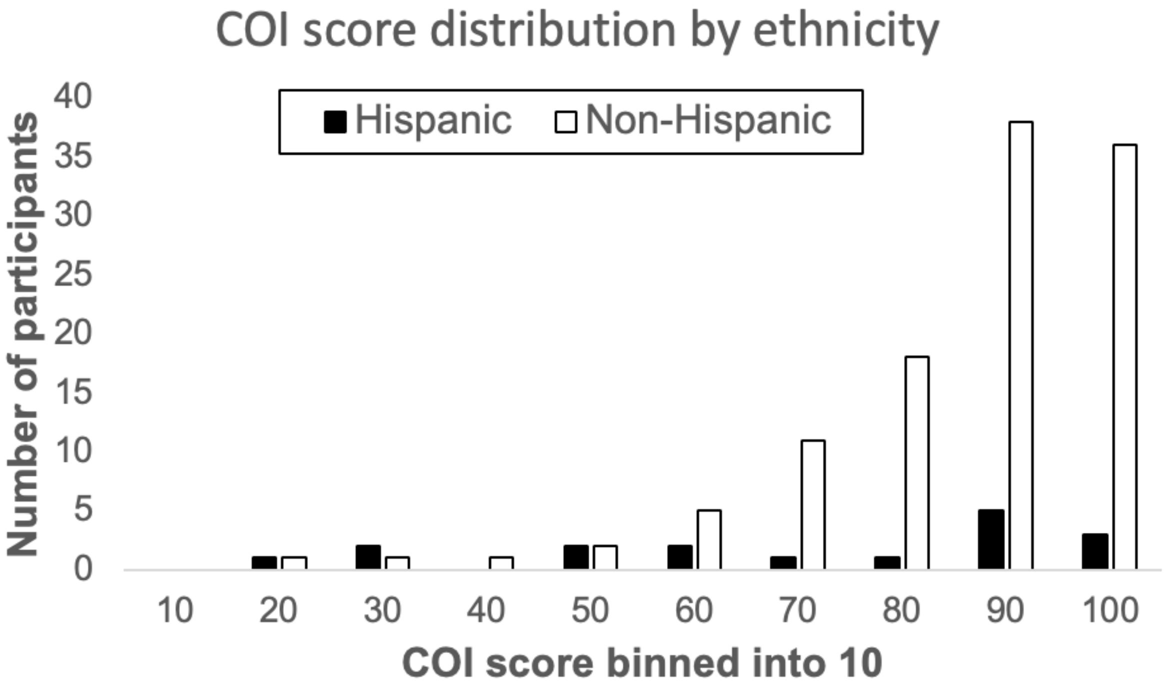

Figure S2. Distribution of the national ranked COI score by ethnicity

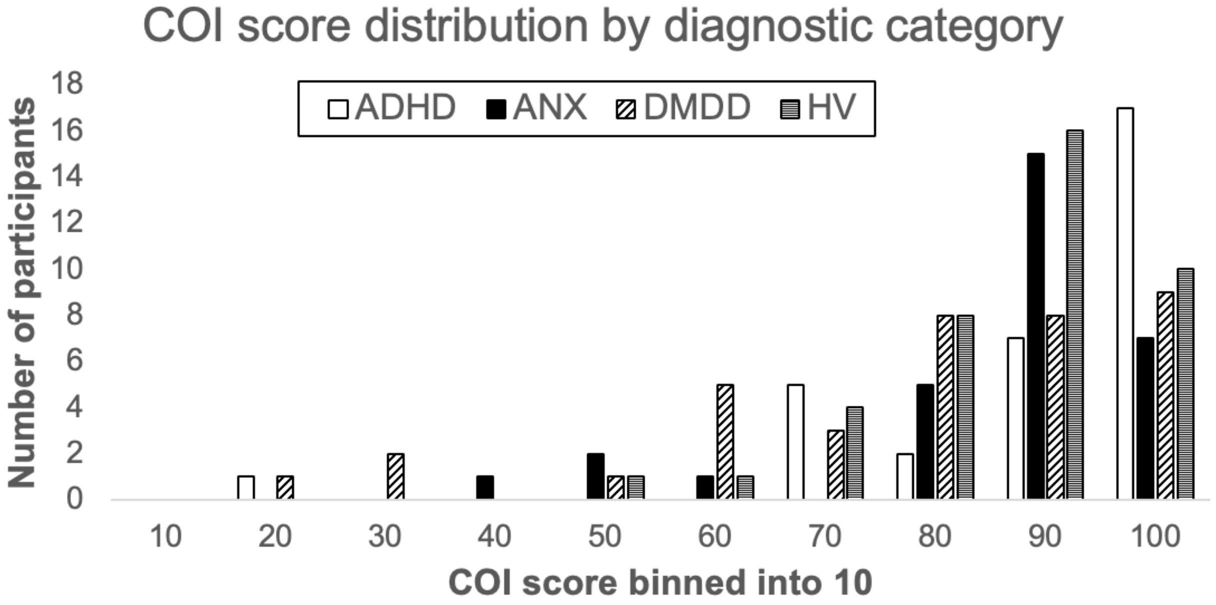

Figure S3. Distribution of the national ranked COI score by diagnostic category

Note: ADHD = Attention-Deficit/Hyperactivity Disorder, **ANX** = Anxiety Disorder, **DMDD** = Disruptive Mood Dysregulation Disorder, **HV** = healthy volunteer, no psychiatric disorder diagnosed

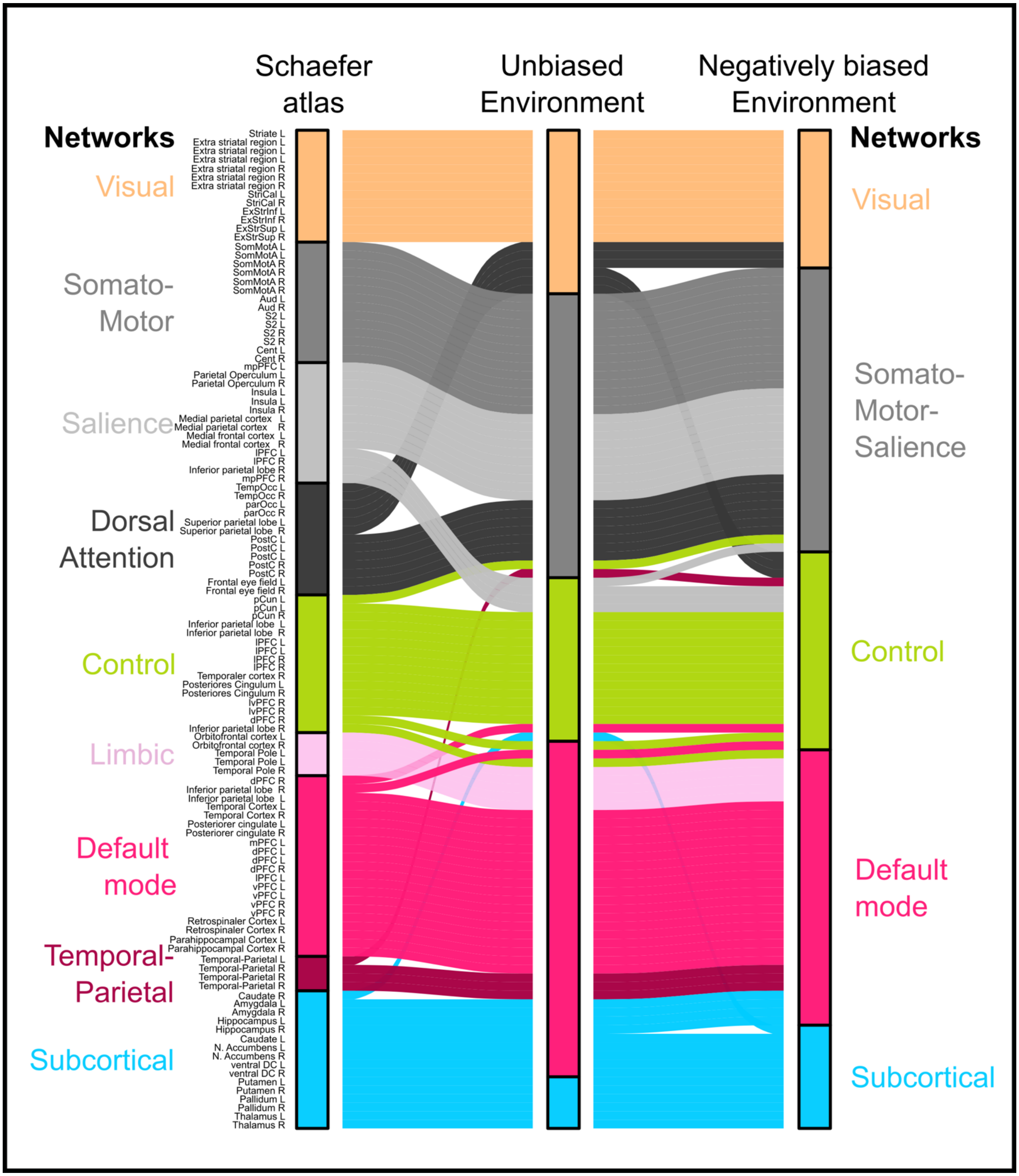

Figure S4. Modular structure and nodal composition of the Schaefer parcellation and in our data during the unbiased and negatively biased condition.

Note: d = dorsal; **l =** lateral; **m =** medial; **PFC** = prefrontal cortex; **v =** ventral

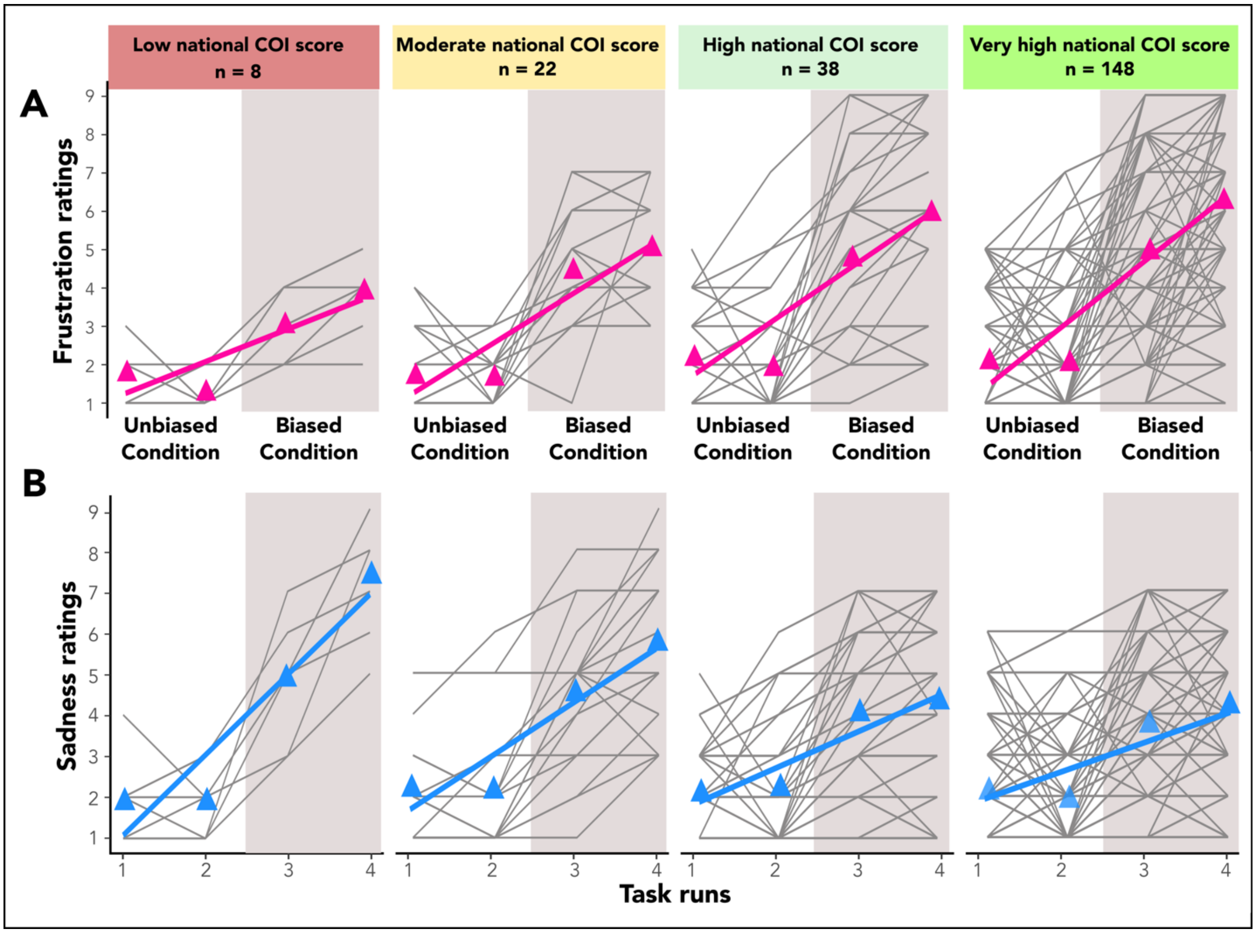

Figure S5. Effect of neighborhood opportunity on frustration (panel A) and sadness (panel B). Gray lines represent the raw data, and colored lines show the median effect of the respective COI group defined relative to the US. Note that we had only one participant living in a neighborhood with "very low" opportunity relative to all >72,000 neighborhoods in the US. Therefore, data from individuals in "low" and "very low" opportunity neighborhoods are grouped together.

Table S2. Comparison between the basic model explaining variance in frustration ratings and models including the COI-score or one of the three subdomain scores

	npar	AIC	BIC	logLik	deviance	X^2	df	p-value
Basic	5	3345.1	3368.9	−1667.6	3335.1			
COI score	8	3331.1	3369.1	−1657.6	3315.1	20.022	3	<0.001
Social and economic subscore	8	3332.8	3370.8	−1658.4	3316.8	18.327	3	<0.001
Health & Environment subscore	8	3342.5	3380.5	−1663.3	3326.5	8.632	3	0.035
Education subscore	8	3337.9	3375.9	−1661.0	3321.9	13.230	3	0.004

Note: ***AIC* =** Akaike Information Criterion (an estimate of prediction error); ***BIC* =** Bayesian Information Criterion; ***COI* =** Childhood Opportunity Index; ***df* =** degree of freedom; ***logLik* =** Log-Likelihood (an unpenalized goodness of fit measure); ***npar* =** number of parameters

Table S3. Parameter estimates for the basic model and the model, including the COI score predicting task-induced changes in frustration ratings

	Frustration rating			Frustration rating		
Predictors	*Estimates*	*CI*	*p-value*	*Estimates*	*CI*	*p-value*
(Intercept)	−1.2	−1.58 – −0.83	**<0.001**	−0.56	−2.24 – 1.12	0.511
Game	3.2	3.00 – 3.40	**<0.001**	1.78	0.84 – 2.72	**<0.001**
Cohort	0.03	−0.20 – 0.25	0.823	0.06	−0.16 – 0.28	0.587
COI score				−0.01	−0.03 – 0.01	0.384
Game × COI score				0.02	0.01 – 0.03	**0.002**
**Random Effects**						
σ^2	2.2			2.17		
τ_{00}	1.36 $_{\text{participant}}$			1.22 $_{\text{participant}}$		
				0.26 $_{\text{Micro areas}}$		
ICC	0.38			0.4		
N	212 $_{\text{participant}}$			22 $_{\text{Micro areas}}$		
				212 $_{\text{participant}}$		
Observations	848			848		
Marginal R^2 / Conditional R^2	0.419 / 0.641			0.424 / 0.657		

Note: ***CI* =** confidence interval; ***COI* =** Childhood Opportunity Index; ***ICC* =** intraclass correlation coefficient; ***N* =** number

Table S4. Comparison between the basic model explaining variance in sadness ratings and models including the COI-score or one of the three subdomain scores

	npar	AIC	BIC	logLik	deviance	X^2	df	p-value
Basic	5	3106.3	3130.0	−1548.2	3096.2			
COI score	8	3080.2	3118.2	−1532.1	3064.2	32.057	3	<0.001
Social and economic subscore	8	3083.4	3121.3	−1533.7	3067.4	28.903	3	<0.001
Health & Environment subscore	8	3097.0	3135.0	−1540.5	3081.0	15.273	3	0.002
Education subscore	8	3088.4	3126.2	−1536.2	3072.4	23.909	3	<0.001

Note: ***AIC* =** Akaike Information Criterion (an estimate of prediction error); ***BIC* =** Bayesian Information Criterion; ***COI* =** Childhood Opportunity Index; ***df* =** degree of freedom; ***logLik* =** Log-Likelihood (an unpenalized goodness of fit measure); ***npar* =** number of parameters

Table S5. Parameter estimates for the basic model and the model, including the COI score predicting task-induced changes in sadness ratings

	Sadness rating			Sadness rating		
Predictors	*Estimates*	*CI*	*p-value*	*Estimates*	*CI*	*p-value*
(Intercept)	−0.04	−0.38 – 0.29	0.792	−2.21	−3.64 – −0.78	**0.003**
Game	2.16	1.99 – 2.33	**<0.001**	4.21	3.42 – 4.99	**<0.001**
Cohort	0.13	−0.08 – 0.34	0.224	0.12	−0.09 – 0.33	0.278
COI score				0.03	0.01 – 0.04	**0.003**
Game × COI score				−0.02	−0.03 – −0.02	**<0.001**
**Random Effects**						
σ^2	1.58			1.52		
τ_{00}	1.27 participant			1.25 participant		
				0.00 Micro areas		
ICC	0.45					
N	212 participant			22 Micro areas		
				212 participant		
Observations	848			848		
Marginal R^2 / Conditional R^2	0.293 / 0.608			0.455 / 0.624		

Note: ***CI* =** confidence interval; ***COI* =** Childhood Opportunity Index; ***ICC* =** intraclass correlation coefficient; ***N* =** number

Table S6. Comparison between the basic model explaining modularity in the biased condition and models including the COI-score or one of the three subdomain scores

	npar	AIC	BIC	logLik	deviance	X^2	df	p-value
Basic	6	−829.5	−809.4	420.8	−841.5			
COI score	7	−831.7	−808.2	422.9	−845.7	4.233	1	0.040
Social and economic subscore	7	−830.7	−807.2	422.4	−848.7	3.216	1	0.073
Health & Environment subscore	7	−831.5	−808.0	422.7	−845.5	3.971	1	0.046
Education subscore	7	−832.2	−808.7	423.1	−846.2	4.739	1	0.029

Note: ***AIC =*** Akaike Information Criterion (an estimate of prediction error); ***BIC =*** Bayesian Information Criterion; ***COI =*** Childhood Opportunity Index; ***df =*** degree of freedom; ***logLik =*** Log-Likelihood (an unpenalized goodness of fit measure); ***npar =*** number of parameters

Table S7. Parameter estimates for the basic model and the model, including the COI score predicting modularity when the invalid cue is presented during the biased condition

	Modularity (Q)			Modularity (Q)		
Predictors	*Estimates*	*CI*	*p-value*	*Estimates*	*CI*	*p-value*
(Intercept)	0.12	0.11 – 0.13	**<0.001**	0.14	0.12 – 0.17	**<0.001**
Cohort	0	−0.00 – 0.01	0.221	0	−0.00 – 0.01	0.279
Mean framewise displacement	−0.03	−0.04 – −0.01	**0.003**	−0.03	−0.04 – −0.01	**0.004**
Scanner	0	−0.01 – 0.01	0.984	0	−0.01 – 0.01	0.91
COI score				0	−0.00 – −0.00	**0.042**
**Random Effects**						
σ^2	0			0		
τ_{00}	0.00 micro areas			0.00 micro areas		
N	22 micro areas			22 micro areas		
Observations	212			212		
Marginal R^2 / Conditional R^2	0.058 / 0.058			0.076 / 0.076		

Note: ***CI* =** confidence interval; ***COI* =** Childhood Opportunity Index; ***ICC* =** intraclass correlation coefficient; ***N* =** number

Table S8. Comparison between the basic model explaining functional connectivity between the somatomotor salience and the control network during the biased condition and models including the COI-score or one of the three subdomain scores

	npar	AIC	BIC	logLik	deviance	X^2	df	p-value
Basic	6	−848.9	−828.8	430.4	−860.9			
COI score	7	−851.7	−826.2	433.5	−864.7	4.169	1	0.041
Social and economic subscore	7	−850.8	−827.4	432.4	−864.8	3.961	1	0.046
Health & Environment subscore	7	−847.4	−823.9	430.7	−861.4	0.521	1	0.470
Education subscore	7	−849.0	−825.5	431.5	−862.9	2.046	1	0.152

Note: ***AIC* =** Akaike Information Criterion (an estimate of prediction error); ***BIC* =** Bayesian Information Criterion; ***COI* =** Childhood Opportunity Index; ***df* =** degree of freedom; ***logLik* =** Log-Likelihood (an unpenalized goodness of fit measure); ***npar* =** number of parameters

Table S9. Parameter estimates for the basic model and the model, including the COI score predicting functional connectivity between the somatomotor salience and the control network during the biased condition

	CON-SOM FC			CON-SOM FC		
Predictors	*Estimates*	*CI*	*Predictors*	*Estimates*	*CI*	*Predictors*
(Intercept)	0.06	0.05 – 0.07	**<0.001**	0.04	0.02 – 0.06	**<0.001**
Cohort	0	−0.00 – 0.01	0.406	0	−0.00 – 0.01	0.331
Mean framewise displacement	0	−0.01 – 0.02	0.723	0	−0.01 – 0.02	0.82
Scanner	0	−0.00 – 0.01	0.316	0	−0.00 – 0.01	0.358
COI score				0	−0.00 – 0.00	**0.038**
**Random Effects**						
σ^2	0			0		
τ_{00}	0.00 micro areas			0.00 micro areas		
N	22 micro areas			22 micro areas		
Observations	211			211		
Marginal R^2 / Conditional R^2	0.024 / 0.024			0.041 / 0.024		

Note: ***CI* =** confidence interval; ***COI* =** Childhood Opportunity Index; ***CON* =** Control network; ***FC* =** functional connectivity; ***ICC* =** intraclass correlation coefficient; ***N* =** number; ***SOM* =** somatomotor salience network
